# Analysis of fatty acids concentration in the liver of rats with diet-induced obesity after bariatric surgery and a high-fat/high-sugar diet

**DOI:** 10.3389/fphys.2026.1784452

**Published:** 2026-04-29

**Authors:** Wojciech Kazura, Katarzyna Michalczyk, Bronisława Skrzep-Poloczek, Marek Michalski, Jerzy Jochem, Elżbieta Chełmecka, Ewa Kaczmar, Jakub Poloczek, Michał Kukla, Dominika Stygar

**Affiliations:** 1 Department of Physiology, Faculty of Medical Sciences in Zabrze, Medical University of Silesia, Katowice, Poland; 2 Department of Histology and Cell Pathology, Faculty of Medical Sciences in Zabrze, Medical University of Silesia, Katowice, Poland; 3 Department of Statistics, Faculty of Pharmaceutical Sciences in Sosnowiec, Medical University of Silesia, Katowice, Poland; 4 Department of Instrumental Analysis, Faculty of Pharmaceutical Sciences in Sosnowiec, Medical University of Silesia, Katowice, Poland; 5 University of Warmia and Mazury in Olsztyn, Olsztyn, Poland; 6 Medical University of Silesia, Katowice, Poland; 7 Department of Internal Medicine and Geriatrics, Faculty of Medicine, Jagiellonian University Medical College, Kraków, Poland; 8 Department of Endoscopy, University Hospital in Kraków, Kraków, Poland

**Keywords:** bariatric surgery, diet-induced obesity, duodenojejunal omega switch, fatty acids, gas chromatography-mass spectrometry, high-fat/high-sugar diet, lipid metabolism, Sprague-Dawley rat

## Abstract

**Introduction:**

Obesity is a leading risk factor for metabolic diseases, but the effects of bariatric surgery on hepatic fatty acid profiles remain poorly understood. This study investigated how duodenojejunal omega switch (DJOS) bariatric surgery and dietary patterns alter fatty acid composition in the liver of rats with diet-induced obesity.

**Methods:**

Fifty-six male Sprague-Dawley rats were fed either a control or a high-fat/high-sugar diet, then assigned to DJOS or SHAM surgery, followed by maintenance or a switch in dietary regimen for eight weeks postoperatively. Liver fatty acids were quantified using gas chromatography-mass spectrometry.

**Results and discussion:**

DJOS surgery combined with a high-fat/high-sugar diet led to significantly higher concentrations of select long- and very-long-chain fatty acids (C14:0, C15:0, C22:0, C24:1, C24:0) versus SHAM, while dietary change post-surgery (CD/CF) reduced concentrations of C17:0, C18:0, C20:4n6, C20:5n3, C22:n6-3, and C24:0 in SHAM-operated rats. Additionally, lower levels of a total mixture of C18:1n9c, C18:2n6t, and C18:3n3 were observed when diet quality improved after SHAM surgery. The findings highlight complex interactions between surgical intervention and dietary composition in modulating hepatic fatty acid metabolism, emphasizing the importance of both approaches in obesity-related metabolic management.

## 1 Introduction

Obesity is an overwhelming and escalating issue in both industrialized and developing nations. Forty years ago, the prevalence of obesity in most high-income nations ranged from less than 5% to 10% (Sattar et al., 2024). Presently, the increased accessibility of high-calorie, low-nutrient meals, especially those abundant in simple sugars and saturated fats, alongside a more sedentary lifestyle, results in over 60% of European adults and nearly one in three children being affected by overweight and obesity (WHO Regional Office for Europe, 2022). This directly contributes to the progression of prevalent chronic diseases, such as type 2 diabetes, hypertension, atherosclerosis, and non-alcoholic fatty liver disease, which pose a severe burden on the public health system (Welsh et al., 2024).

Pathophysiological alterations characteristic of obesity and its related metabolic syndrome, including hyperglycemia and elevated lipid levels, lead to the generation of reactive oxygen species, disrupting the equilibrium between pro- and antioxidant mechanisms. This results in diminished antioxidant defense, widespread chronic inflammation, and heightened susceptibility to numerous severe diseases, including those affecting the cardiovascular system and cancer. Currently, obesity treatment is based on three pillars. The basis of therapy is lifestyle strategies, including maintaining a negative caloric balance by reducing dietary intake and increasing physical activity (Ryan, 2023). The second aspect of treatment is pharmacotherapy, which has made significant progress, especially in recent years, using GLP-1 and GIP analogues (Melson et al., 2023).

Although studies have shown that already a 10% weight loss has a positive clinical effect (Tahrani and Morton, 2022), in patients with morbid obesity, greater weight loss may be necessary to improve or achieve remission of some obesity-related complications and reduce the risk of cardiovascular events. In patients who have failed to achieve and maintain satisfactory weight loss with other methods, bariatric surgery may be an option (Wolfe et al., 2016).

Bariatric surgery can be divided into three main types: restrictive surgery (which aims to significantly reduce the capacity of the stomach, thereby limiting the amount of food intake), malabsorptive/exclusive surgery (which causes a restriction of the proper absorption of nutrients), and combined surgery (which combines restrictive and malabsorptive elements) (Park and Jun, 2025).

Bariatric surgery is not only an effective method of reducing excess body weight, but it also has beneficial metabolic effects by influencing mechanisms regulating carbohydrate and lipid homeostasis, thereby reducing insulin resistance, improving diabetes control, and improving lipid profile (Różańska-Walędziak et al., 2024).

Duodenal-jejunal omega switch (DJOS) is a type of bariatric procedure that involves the formation of a close duodenal-jejunal loop that excludes the foregut and allows direct activation of the hindgut. The advantage of this procedure is that it preserves the pylorus of the stomach. Such modification of the gastrointestinal tract prevents undesirable symptoms associated with post-gastrectomy conditions such as postprandial syndrome, diarrhea, or dyspepsia. This metabolic surgical operation has been shown to have beneficial effects on body weight, glucose tolerance, redox status, and hormonal signaling via hepatokines and adipokines (Stygar et al., 2019a; Poloczek et al., 2021; Poloczek et al., 2022). However, little is known about the effects of this procedure on lipid composition, especially on fatty acids concentrations derived from polar and non-polar lipids.

Fatty acids (FAs) are a major nutritional component of food and play a crucial role in maintaining health. These organic molecules are not only responsible for energy supply and storage but also form structural elements in cell membranes (polar lipids) and serve as precursors of various bioactive lipid mediators (Casares et al., 2019). Fatty acids are a complex group divided into different classes based on their chemical structure and the type of chemical bonds. Saturated fatty acids (SFAs) have single bonds only in their hydrocarbon chains, making them relatively stable and solid at room temperature. Although SFAs perform important physiological functions, excessive intake triggers an inflammatory response in tissues and is associated with metabolic syndrome (Ravaut et al., 2021). In opposition, unsaturated fatty acids (UFAs), having one (monounsaturated fatty acids (MUFAs)) or multiple (polyunsaturated fatty acids (PUFAs)) double bonds in their structure, are valued for their potential health benefits, since they have been shown to improve insulin sensitivity, as well as lessen inflammation (Wall et al., 2010). The Mediterranean diet is known to be rich in unsaturated fatty acids, as they are abundant in olive oil, avocados, nuts, and marine fish (Liu et al., 2023). The presence of multiple double bonds in PUFAs increases their polarity, which can affect their interactions with cellular membranes and various enzymes, membrane fluidity, cell signaling, and eicosanoid production (Kapoor et al., 2021).

The study analyzes the content and composition of FAs in rat liver tissue, which is the most common animal model used in lipid metabolism research. Moreover, it aims to identify potential functional biomarkers of obesity-related propensity and to analyze the effect of bariatric surgery on lipid metabolism in relation to obesity, including diet-induced obesity, using selected dietary patterns. The experimental design accounts for clinical observations that not all patients maintain a caloric regimen after bariatric surgery and considers two additional situations: when some patients switch from a normal to a high-fat/high-sugar diet, and from a high-fat/high-sugar diet to a normal diet.

## 2 Methods

### 2.1 Ethical permissions

The procedures within the experimental design were approved by the Ethical Committee for Animal Experimentation of the Medical University of Silesia (Katowice, Poland) (58/2014). The institutional and national guidelines for animal care and use (Directive 2010/63/EU) were followed. The number of animals used in the study was minimal, as the 3Rs guidelines for the humane treatment of animals suggest (Russell and Burch, 1959). The survival rate of the animals undergoing surgery was 100%.

### 2.2 Study subject

The study included 56 7-week-old male Sprague-Dawley rats (Charles River Breeding Laboratories, Wilmington, MA, USA) weighing 250–275 g. Male Sprague-Dawley rats were used as the experimental model to limit biological variability associated with the estrous cycle. Fluctuations in ovarian hormones, particularly estrogen and progesterone, significantly affect food intake, body weight regulation, adipose tissue distribution, insulin sensitivity, and lipid metabolism of females, thereby increasing intra-group variability and complicating the statistical interpretation of results. Moreover, male rodents typically exhibit more pronounced and reproducible metabolic responses to a high-fat diet, including greater body weight gain, visceral fat accumulation, dyslipidemia, and insulin resistance, which facilitates the detection of diet- or intervention-induced effects (Hwang et al., 2010; Salinero et al., 2018).

The animals were kept in plastic cages containing 3–4 individuals, in fully controlled conditions at 23 °C with a 12-h light/dark cycle. Access to food and water for the animals during the entire experiment was unlimited, ad libitum. For this reason, the individual food intake data were not collected. Instead, the body weight of each individual was recorded regularly throughout the experiment.

### 2.3 Study design

After a 7-day acclimation to laboratory conditions during which a control diet was used, the animals were randomly divided into the experimental groups. The control group (n = 28) was maintained on a normal diet (Provimi Kliba AG, Kaiseraugst, Switzerland, 24% protein, 4.9% fat, 7% crude ashes, 4.7% crude fibre). Obesity was induced by keeping the animals on a high-fat/high-sugar diet (CF) (*n* = 28) (22.0 MJ/kg, 45% fat, 35% carbohydrate and 20% protein (ssniff^®^ EF R/M acc. D12451 (II) mod., Ssniff Spezialdiäten GmbH, Germany) for 8 weeks before and after the surgery.

**Figure 1:**
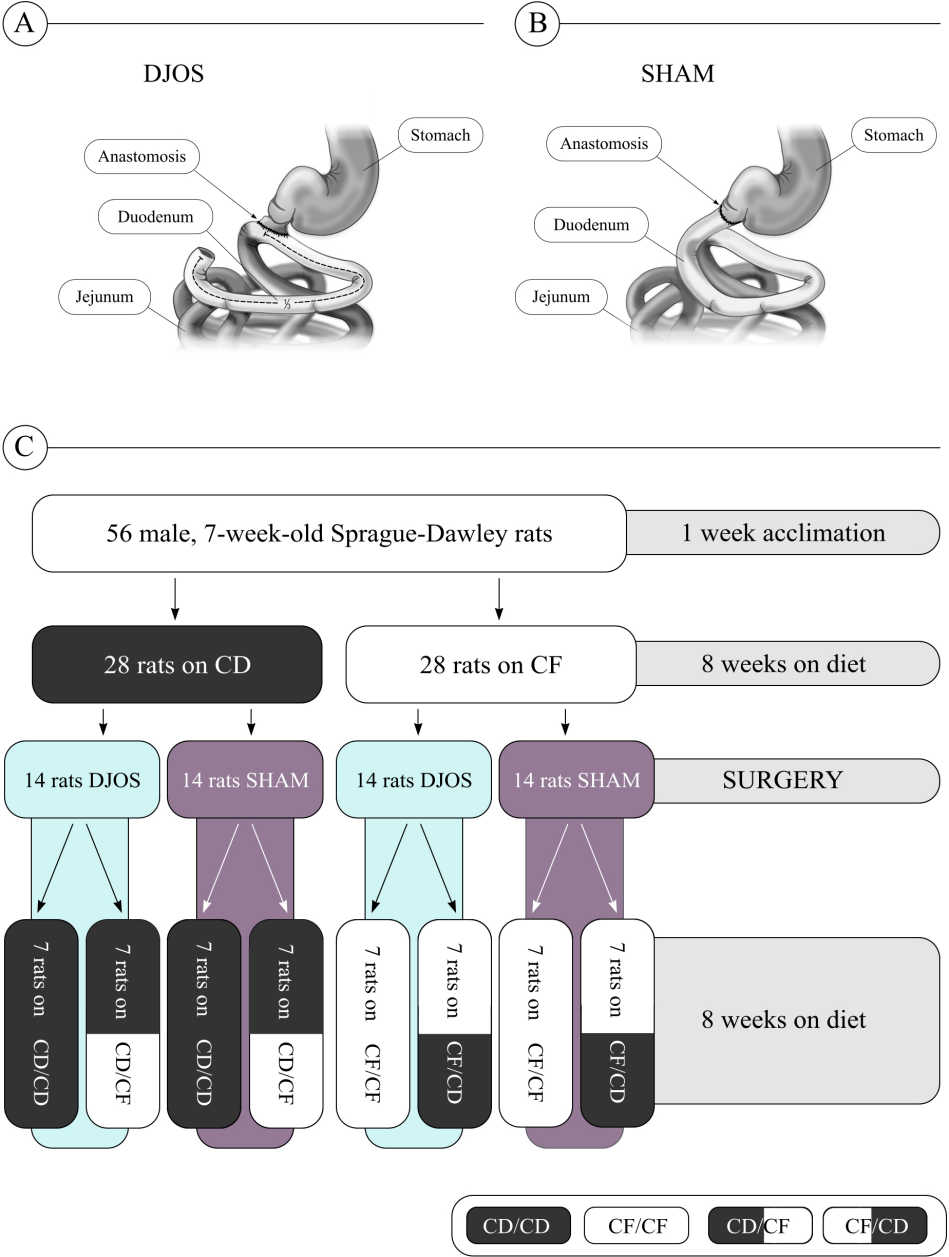
Study design and experimental groups of Sprague-Dawley rats subjected to **(A)** duodenojejunal omega switch (DJOS) surgery or **(B)** control (SHAM) surgery, and **(C)** different dietary protocols. Legend: CD, control diet; CF, high-fat/high-sugar diet.

After 8 weeks, rats from each experimental group (CD and CF) were randomly subjected to either control (SHAM) (n = 14) or duodenojejunal omega switch (DJOS) (n = 14) surgery, creating four subgroups: CD/SHAM, CD/DJOS, CF/SHAM, and CF/DJOS. After the surgery, rats from each subgroup were fed either the same diet or a changed diet for an additional 8 weeks, creating eight final cohorts of 7 individuals (**Figure 1**).

### 2.4 Experimental procedures

#### 2.4.1 Control (SHAM) and duodenojejunal omega switch (DJOS) surgery

All rats fasted overnight before surgery.

The animals were subjected to anesthesia with 2% isoflurane (AbbVie, Ludwigshafen, Germany) and oxygen 2 L/min under spontaneous breathing, analgesia with xylazine (5 mg/kg, ip; Xylapan, Vetoquinol Biovet, Puławy, Poland), and antibiotic prophylaxis with gentamicin (gentamycin 40 mg/mL, Krka, Warsaw, Poland).

The abdominal cavity was accessed through a 3–4 cm midline incision. The duodenum was separated from the stomach at the point just below the pyloric sphincter. For DJOS, a new end-to-side anastomosis (duodeno-enterostomy) was placed at one-third of the total length of the small bowel to restore intestinal passage and exclude the entire duodenum and the proximal jejunum. The excluded duodenum was sutured using PDS 6/0 (Ethicon, Cincinnati, OH, USA). The mesentery was also closed with PDS 6/0.

During the control procedure (SHAM), transections and re-anastomosis of the gastrointestinal tract were performed at the corresponding sites where enterotomies were performed for the DJOS surgery, thereby maintaining the physiological conduit of food passage through the bowel.

The analgesia was maintained for 72 h after the surgery with carprofen (4 mg/kg, sc; Rimadyl, Pfizer, Zürich, Switzerland).

#### 2.4.2 Sample collection and preparation

The animals were euthanized according to the procedures approved by the Ethical Committee for Animal Experimentation of the Medical University of Silesia (Katowice, Poland). The animals were subjected to anesthesia with 2% isoflurane (AbbVie, Ludwigshafen, Germany) and oxygen 2 L/min under spontaneous breathing and analgesia with xylazine (5 mg/kg, ip; Xylapan, Vetoquinol Biovet, Puławy, Poland), and euthanized with 100 mg/kg pentobarbital injection (Morbital, 133.3 mg/mL sodium pentobarbital + 26.7 mg/mL pentobarbital, Biowet Puławy Sp. z o.o., Puławy, Poland).

The lobe of the liver was removed after opening the abdominal cavity immediately after euthanasia. The liver tissues weighing ~100 mg were collected in 0.9% NaCl, and 10% homogenates were prepared.

The extracting mixture (0.5 mL, hexane: isopropanol, 3:2 vol./vol) was added to the liver homogenates. Homogenates were shaken using a mechanical shaker (800 rpm, 20 min) at room temperature, and subsequently centrifuged at 10–000 *g* for 10 min. The supernatant (organic phase) was transferred to a clean Eppendorf tube. The 0.5 mL of Na_2_SO_4_ (0.1 M) was added to the supernatant, then vigorously shaken, and centrifuged again (10–000 *g* for 10 min). Lipid-containing supernatant was placed in a clean Eppendorf tube and dried on a thermoblock under a low-pressure nitrogen atmosphere (39 °C, 15 min).

Nitrogen-dried lipid samples were transesterified according to Christie’s method (Christie, 1987; Christie, 2003).

Lipid samples were dissolved in 1 mL of toluene, and after vortexing, 2 mL of a methanol-sulphuric acid (20 mL/L) mixture was added. The samples were then incubated in a thermoblock (50 °C, 18 h). After cooling, 2 mL of a mixture of sodium bicarbonate and sodium carbonate was added (0.25 M NaHCO_3_:0.5 M Na_2_CO_3_) followed by 2 mL of hexane. Samples were shaken and centrifuged (10–000 g, 14 °C, 10 min). The supernatant (hexane-toluene phase) was collected in a clean tube and dried on a thermoblock under a low-pressure nitrogen atmosphere (38 °C, 15 min). The lipid samples were stored at −80 °C until further analysis.

#### 2.4.3 Fatty Acid Methyl Esters determinations by GC-MS technique

Before analysis, the collected lipid samples were dissolved in 250 µL of toluene, vortexed for 2 min, and then sonicated in an ultrasonic bath for 5 min at room temperature. The samples were then centrifuged using a laboratory centrifuge for 4 min at 4–000 rpm at room temperature. Fatty Acid Methyl Esters (FAME) were assessed using a gas chromatograph (Trace 1310 Thermo Scientific, Milano, Italy) coupled to a triple quadrupole mass spectrometer (TSQ 9000, San Jose, CA, USA) with electron impact ionization (70 eV). The sample extracts were separated on a TG-5MS GC capillary column (30 m × 0.25 mm ID; 0.25 μm) (Thermo Scientific, Waltham, MA, USA). Helium was used as the carrier gas, with a constant flow rate of 1.5 mL/min. Standard solutions and biological sample extracts in toluene were dosed to the column using an automatic sample feeder in the amount of 1 µl, using the injection technique into the feeder with a flow split of 1:10. The column temperature was programmed to rise from 40 to 300 °C at a rate of 5 °C/min. FAME determinations were performed in the full scan mode of the formed ions (mass range from 50 to 550 Da). The FAME Standard Mixture was used for the identification and quantification of fatty acid profiles (Supelco 37 Component FAME MIX, CRM47885, Sigma Aldrich, Steinheim, Germany). The FAME Standard Mixture and blanks were run every 10 analyses to ensure consistency of the obtained results. The LOD for peaks was set at a 3:1 signal-to-noise ratio, and the LOQ was set at a 3× LOD. Absolute values were read against the calibration curve and expressed as ng/μL of the sample volume. FAME identification consisted of comparing retention times on chromatograms and mass spectra recorded for standards and compounds present in biological sample extracts, using Chromeleon software (version 7.2.10) and the Mainlib and NIST (National Institute of Standards and Technology, USA) library databases. The temperature parameters for FAME determinations were selected based on the literature (Zeng et al., 2013) with minor modifications.

The list of assessed FAs is given in **Table 1**.

**Table 1:** List of identified fatty acids with their numbering, common names, and systematic names, categorized into short-chain, medium-chain, long-chain, very long-chain fatty acids (SCFA, MCFA, LCFA, VLCFA), as well as monounsaturated and polyunsaturated fatty acids (MUFA, PUFA).

Lipid number	ω−n	Common name	Systematic name
Short-chain fatty acid (SCFA)			
C4:0		Butyric acid	Butanoic acid
Medium-chain fatty acids (MCFA)			
C6:0		Caproic acid	Hexanoic acid
C8:0		Caprylic acid	Octanoic acid
C10:0		Capric acid	Decanoic acid
C11:0		Undecylic acid	Undecanoic acid
C12:0		Lauric acid	Dodecanoic acid
Long-chain fatty acids (LCFA)			
C13:0		Tridecylic acid	Tridecanoic acid
C14:0		Myristic acid	Tetradecanoic acid
C15:0		Pentadecylic acid	Pentadecanoic acid
C16:0		Palmitic acid	Hexadecanoic acid
C17:0		Margaric acid	Heptadecanoic acid
C18:0		Stearic acid	Octadecanoic acid
C20:0		Arachidic acid	Icosanoic acid
C21:0		Heneicosylic acid	Heneicosanoic acid
Very long chain fatty acids (VLCFA)			
C22:0		Behenic acid	Docosanoic acid
C23:0		Tricosylic acid	Tricosanoic acid
C24:0		Lignoceric acid	Tetracosanoic acid
Monounsaturated fatty acids (MUFA)			
C14:1	ω−5	Myristoleic acid	9-*cis*-Tetradecenoic acid
C15:1			10-*cis*-Pentadecenoic acid
C16:1	ω−7	Palmitoleic acid	9-*cis*-Hexadecenoic acid
C17:1			10-*cis*-Heptadecenoic Acid
C18:1n9t	ω−9	Elaidic acid	*trans*-9-Octadecenoic acid
C18:1n9c	ω−9	Oleic acid	*cis*-9-Octadecenoic acid
C20:1	ω−9	Gondoic acid	*cis*-11-Eicosenoic acid *cis*-11-Icosenoic acid
C22:1n9	ω−9	Erucic acid	*cis*-13-Docosenoic acid
C24:1(n-9)	ω−9	Nervonic acid	*cis*-15-Tetracosenoic acid
Polyunsaturated fatty acids (PUFA)			
C18:3n3	ω−3	α-Linolenic acid (ALA)	*cis*,*cis*,*cis*-9,12,15-Octadecatrienoic acid
C20:3n3	ω−3	Dihomolinolenic acid	cis,cis,cis-11,14,17-Eicosatrienoic acid
C20:5n3	ω−3	Eicosapentaenoic acid (EPA) Timnodonic acid	*all-cis-*5,8,11,14,17-Eicosapentaenoic acid
C22:6n-3	ω−3	Docosahexaenoic acid (DHA) Cervonic acid	*all-cis-*Docosa-4,7,10,13,16,19-hexaenoic acid
C18:2n6t	ω−6	Linoelaidic acid Linolelaidic acid	*trans*,*trans*-9,12-Octadecadienoic acid
C18:2n6c	ω−6	Linoleic acid (LA)	*cis*,*cis*-9,12-Octadecadienoic acid
C18:3n6	ω−6	γ-Linolenic acid (GLA) Gamolenic acid	*cis,cis,cis*-6,9,12-Octadecatrienoic acid
C20:2n-6	ω−6	Eicosadienoic acid	*cis*-11,14-Eicosadienoic acid
C20:3n6	ω−6	Dihomo-γ-linolenic acid (DGLA)	*cis*,*cis*,*cis*-8,11,14-Eicosatrienoic acid
C20:4n6	ω−6	Arachidonic acid (AA, ARA)	*all*-*cis*-5,8,11,14-Eicosatetraenoic acid
C22:2n6	ω−6	Docosadienoic acid	*cis*-13,16-Docosadienoic acid

### 2.5 Statistical analysis

The statistical analysis included separate analyses for FAs extracted from the liver tissues of DJOS- and SHAM-operated rats. Then, the analysis compared fatty acid concentrations extracted from the liver tissues of rats subjected to different dietary protocols at 8 weeks after surgery.

Statistical analyses were performed using Statistica v. 13.3.0 (TIBCO Software Inc., Palo Alto, CA, USA). All the tests were two-tailed. The normality of distribution was assessed using the Shapiro–Wilk test and quantile–quantile plots. The non-normal data were presented as the median with lower and upper quartiles (Me(Q_1-_Q_3_). The intra-operative comparisons were performed using ANOVA and a non-parametric Kruskal–Wallis test with multiple comparisons. The Mann-Whitney test was used to compare treatment effects (SHAM vs DJOS) within one dietary protocol. The data for the SHAM- and DJOS-operated animals were presented in one graph. Differences at *p* < 0.05 were considered to be statistically significant.

## 3 Results

**Figure 2:**
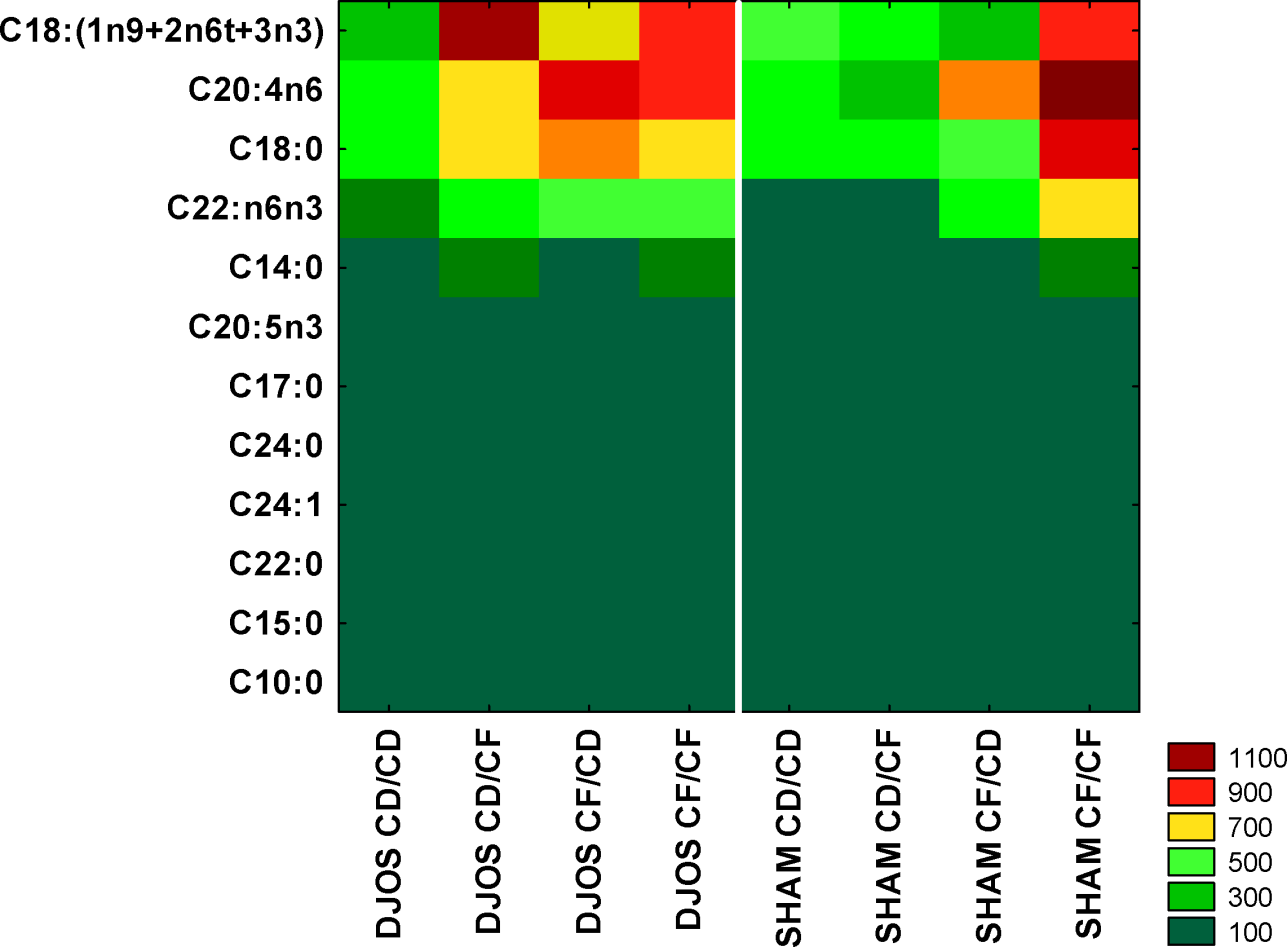
Heatmap of average fatty acid concentrations (ng/μL) in the liver of Sprague-Dawley rats (n = 56) subjected to duodenojejunal omega switch (DJOS, n = 28) surgery or control (SHAM, n = 28) surgery and different dietary protocols (CD/CD, CD/CF, CF/CD, CF/CF, n = 7 for each) at 8 weeks after the surgery. Legend: CD, control diet; CF, high-fat/high-sugar diet. For the reader’s convenience, results for DJOS- or SHAM-operated groups are separated by a white line.

The lowest FAs concentrations were observed both in SHAM and DJOS-operated animals for C10:0, C15:0, C22:0, C24:1, C17:0, and C20:5n3 FAs. The highest concentrations were observed for the total concentration of C18:1n9 + C18:2nt6 + C18:3n3 FAs in the DJOS-operated rats on the CD/CF dietary protocol, whereas in the SHAM-operated rats these values were lower. For C18:0 FA, higher concentrations were observed for the DJOS-operated rats on CD/CF and CF/CD dietary protocols than in SHAM-operated rats. The SHAM-operated rats on the CF/CF dietary protocol showed higher concentrations of C18:0 FA in the liver than the DJOS-operated rats from the corresponding dietary protocol (**Figure 2**).

**Figure 3:**
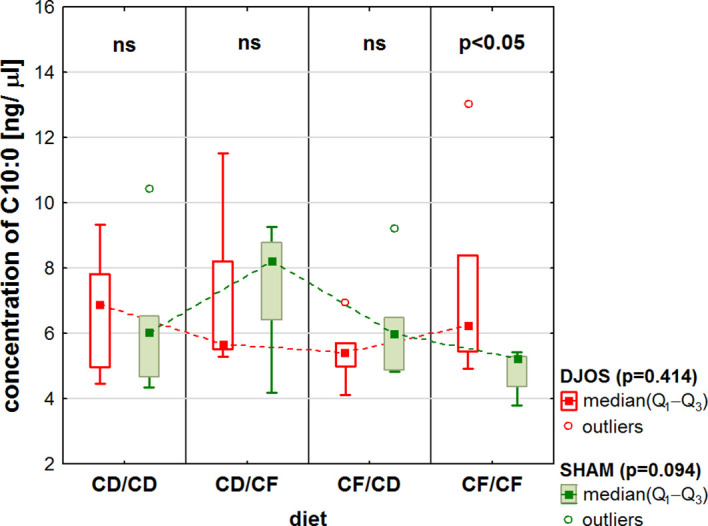
Concentration of C10:0 capronic acid (ng/μL) in the liver of Sprague-Dawley rats (n = 56) subjected to duodenojejunal omega switch (DJOS, n = 28) surgery or control (SHAM, n = 28) surgery and different dietary protocols (CD/CD, CD/CF, CF/CD, CF/CF, n = 7 for each) at 8 weeks after the surgery. Legend: CD, control diet; CF, high-fat/high-sugar diet. Kruskal-Wallis ANOVA was used to compare values across diets, separately for the SHAM and DJOS treatments, and statistical significance values are provided in the legend. Comparisons between treatments within individual diets are presented above the bars. If no differences were observed, ns (not significant) was recorded.

Comparisons of the dietary regimes showed differences in FA concentration in the liver of rats fed with the CF/CF diet (a high-fat/high-sugar diet pre- and post-surgery), depending on the type of operation, for C10:0 capronic acid (MCFA – Medium-Chain Fatty Acids), with higher levels observed in the liver of rats after the DJOS surgery (**Figure 3**).

**Figure 4:**
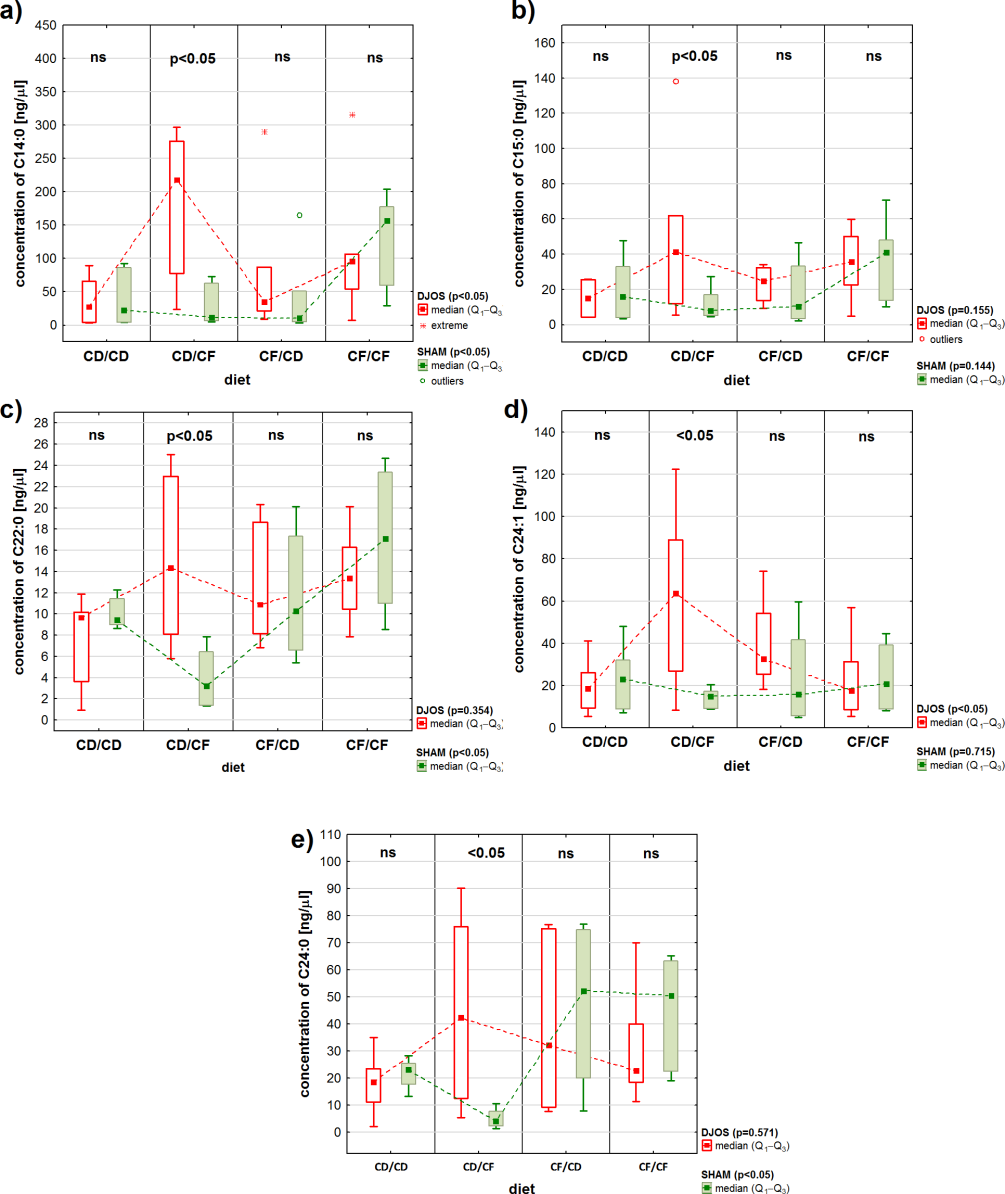
Concentrations (ng/μL) of long-chain fatty acids (LCFA): **(A)** C14:0 myristic acid, **(B)** C15:0 pentadecanoic acid, and very long-chain fatty acids (VLCFA): **(C)** C22:0 behenic acid, **(D)** C24:1 nervonic acid, **(E)** C24:0 lignoceric acid in the liver of Sprague-Dawley rats (n = 56) subjected to duodenojejunal omega switch (DJOS, n = 28) surgery or control (SHAM, n = 28) surgery and different dietary protocols (CD/CD, CD/CF, CF/CD, CF/CF, n = 7 for each) at 8 weeks after the surgery. Legend: CD, control diet; CF, high-fat/high-sugar diet. Kruskal-Wallis ANOVA was used to compare values across diets, separately for the SHAM and DJOS treatments, and statistical significance values are provided in the legend. Comparisons between treatments within individual diets are presented above the bars. If no differences were observed, ns (not significant) was recorded.

The analysis demonstrated differences in 5 fatty acids concentrations, two long-chain fatty acids (LCFA): C14:0 myristic acid and C15:0 pentadecanoic acid, and three very long-chain fatty acids (VLCFA): C22:0 behenic acid, C24:1 nervonic acid, and C24:0 lignoceric acid, in the liver of rats fed with the CD/CF protocol (a control diet pre-surgery and high-fat/high-sugar diet post-surgery), depending on the type of surgery (**Figure 4**). In each case, statistically higher FAs concentrations were observed in rats subjected to DJOS surgery than in rats subjected to SHAM procedure.

**Figure 5:**
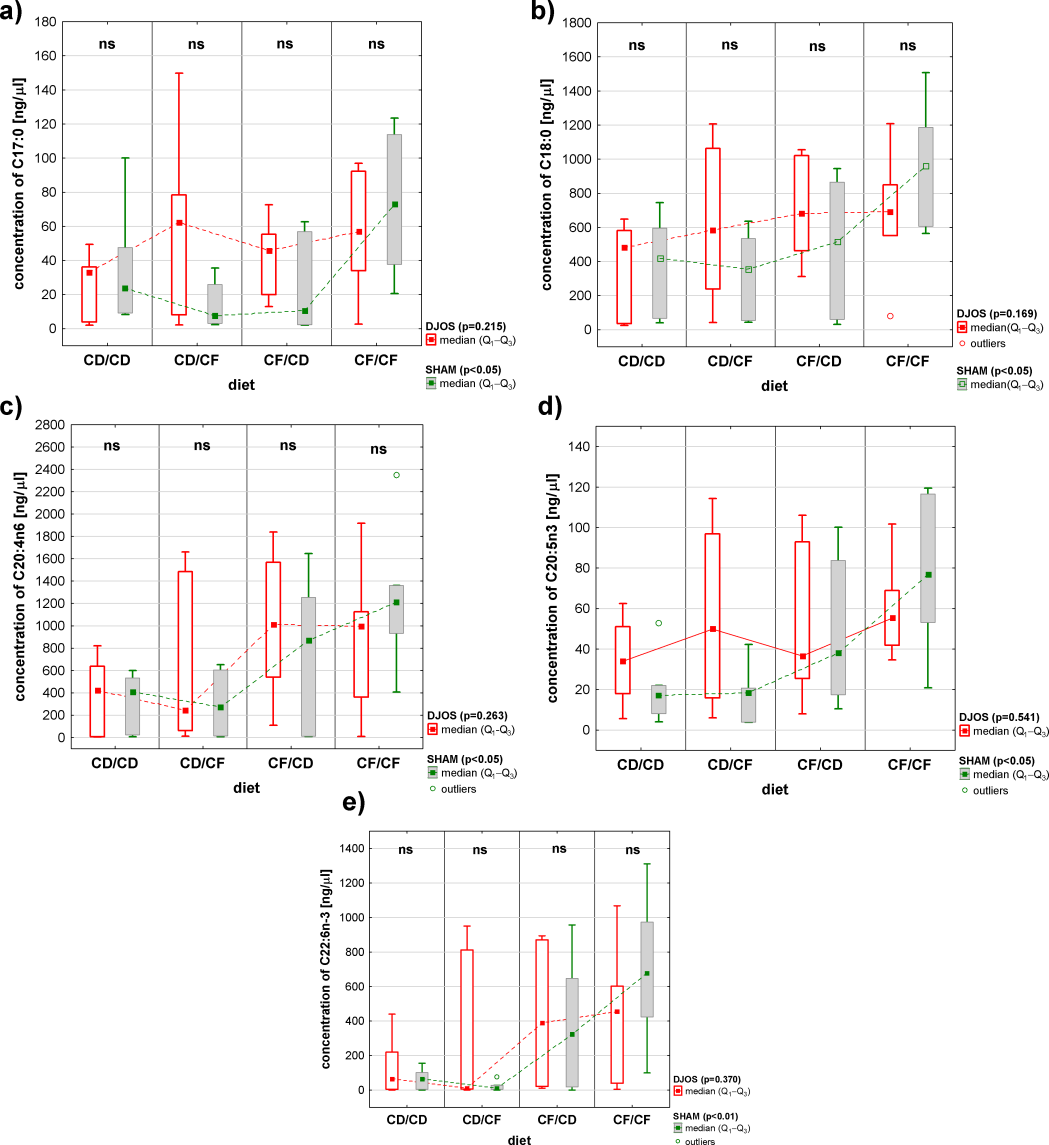
The concentrations (ng/μL) of **(A)** C17:0, **(B)** C18:0, **(C)** C20:4n6, **(D)** C20:5n3, and **(E)** C22:6n-3 fatty acids in the liver of Sprague-Dawley rats (n = 56) subjected to duodenojejunal omega switch (DJOS, n = 28) surgery or control (SHAM, n = 28) surgery and different dietary protocols (CD/CD, CD/CF, CF/CD, CF/CF, n = 7 for each) at 8 weeks after the surgery. Legend: CD, control diet; CF, high-fat/high-sugar diet. Kruskal-Wallis ANOVA was used to compare values across diets, separately for the SHAM and DJOS treatments, and statistical significance values are provided in the legend. Comparisons between treatments within individual diets are presented above the bars. If no differences were observed, ns (not significant) was recorded.

Further analyses showed differences in FAs concentration in the liver of SHAM-operated rats fed with different dietary protocols. Detailed analyses showed significantly lower concentrations of C17:0, C18:0, C20:4n6, C20:5n3, and C22:n6–3 FAs in the liver of rats fed according to the CD/CF dietary protocol than in the liver of rats fed according to the CF/CF dietary protocol (**Figure 5**).

In the case of C24:0 FA, the analysis also demonstrated differences between the rats fed with the CD/CF dietary protocol and those fed with the CF/CD dietary protocol (a high-fat/high-sugar diet pre-surgery and control diet post-surgery) (**Figure 4**).

**Figure 6:**
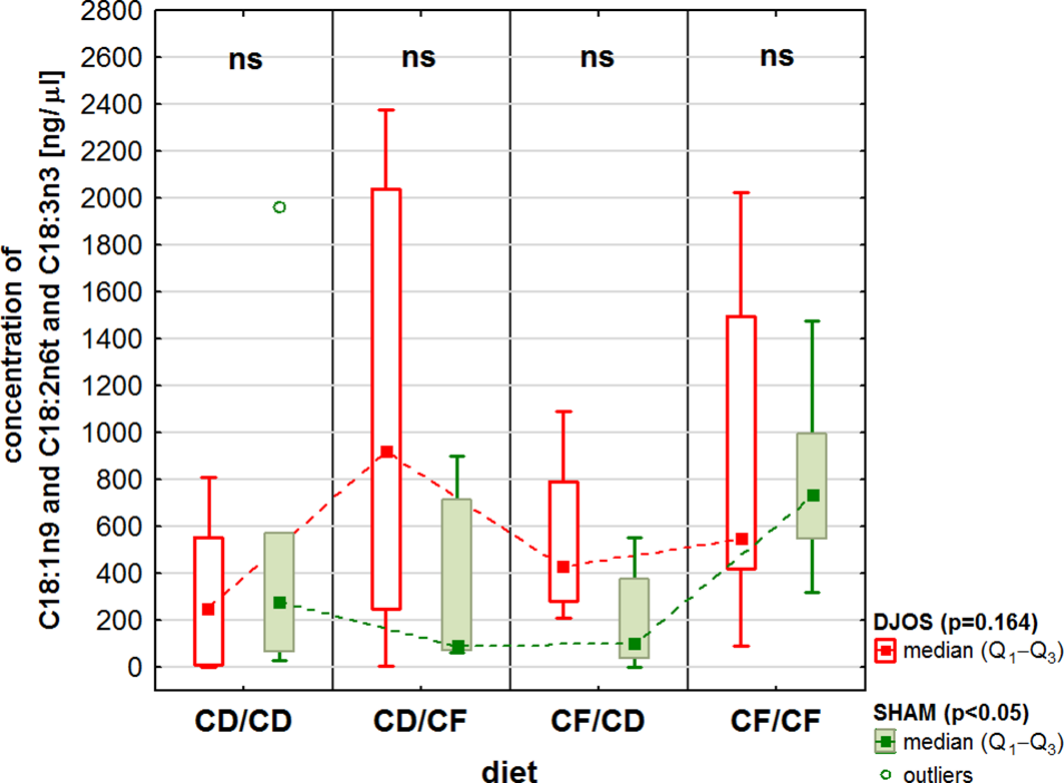
The total acid concentration (ng/μL) for the mixture of C18:1n9c, C18:2n6t, and C18:3n3 fatty acids acid in the liver of Sprague-Dawley rats (n = 56) subjected to duodenojejunal omega switch (DJOS, n = 28) surgery or control (SHAM, n = 28) surgery and different dietary protocols (CD/CD, CD/CF, CF/CD, CF/CF, n = 7 for each) at 8 weeks after the surgery. Legend: CD, control diet; CF, high-fat/high-sugar diet. Kruskal-Wallis ANOVA was used to compare values across diets, separately for the SHAM and DJOS treatments, and statistical significance values are provided in the legend. Comparisons between treatments within individual diets are presented above the bars. If no differences were observed, ns (not significant) was recorded.

As for the total acid concentration for the mixture of C18:1n9c, C18:2n6t, and C18:3n3, the analysis revealed statistically significant differences between SHAM-operated rats fed with the CF/CF dietary protocol and the CF/CD dietary protocol, with lower FAs concentrations after improving the diet postoperatively than in the case of a high-fat/high-sugar diet both before and after the procedure (**Figure 6**).

In the case of other FAs, the analysis showed no statistically significant differences in response to either the type of surgery or the dietary protocol used.

We also assessed the effects of diet and surgical intervention on the animal’s body weight. During the acclimation week, we observed no differences related to allocation to subsequent surgical procedures (p = 0.715) or dietary group (p = 0.176). The mean body weight of rats during the acclimation period was 254.7 ± 2.4 g.

**Figure 7:**
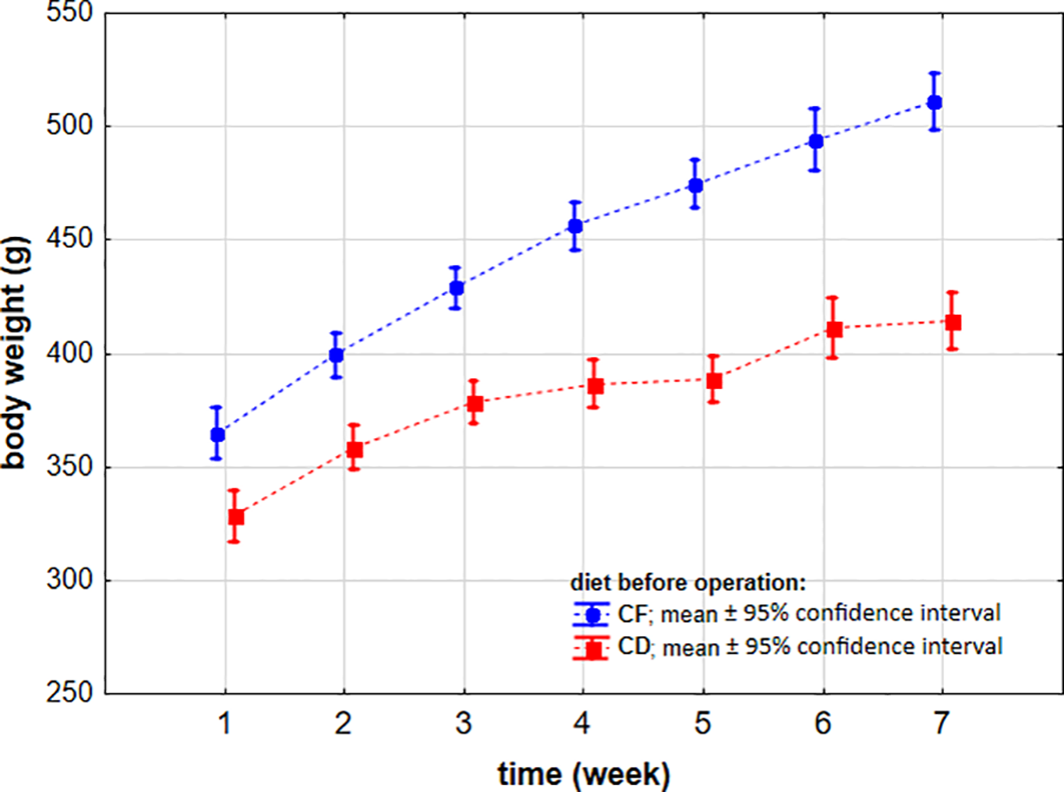
Body weight of Sprague-Dawley rats (n = 56) subjected to different dietary protocols (n = 28 for CD and n = 28 for CF) during the before-surgery (duodenojejunal omega switch (DJOS) or control (SHAM)) phase of the experiment. Legend: CD, control diet; CF, high-fat/high-sugar diet. To compare body weights by diet type, a two-way ANOVA for repeated measures with multiple comparisons analysis was used.

Starting from week 1 to week 7, rats were fed different diets. We observed significant differences in rats’ body weight depending on the dietary protocol (p < 0.001). In each individual week, rats fed the high-fat/high-sugar diet (CF) achieved higher body weights compared with those fed the control diet (CD). Ultimately, in week 7, rats’ body weights reached 414.4 ± 20.3 g in the CD group and 511.1 ± 42.7 g in the CF group (**Figure 7**).

In the subsequent stage of the study, rats were reassigned and subjected to either SHAM or DJOS surgery. We observed significant effects on body weight depending on the dietary protocol (p < 0.001), surgical procedure (p < 0.001), and time since surgery (p < 0.001).

**Figure 8:**
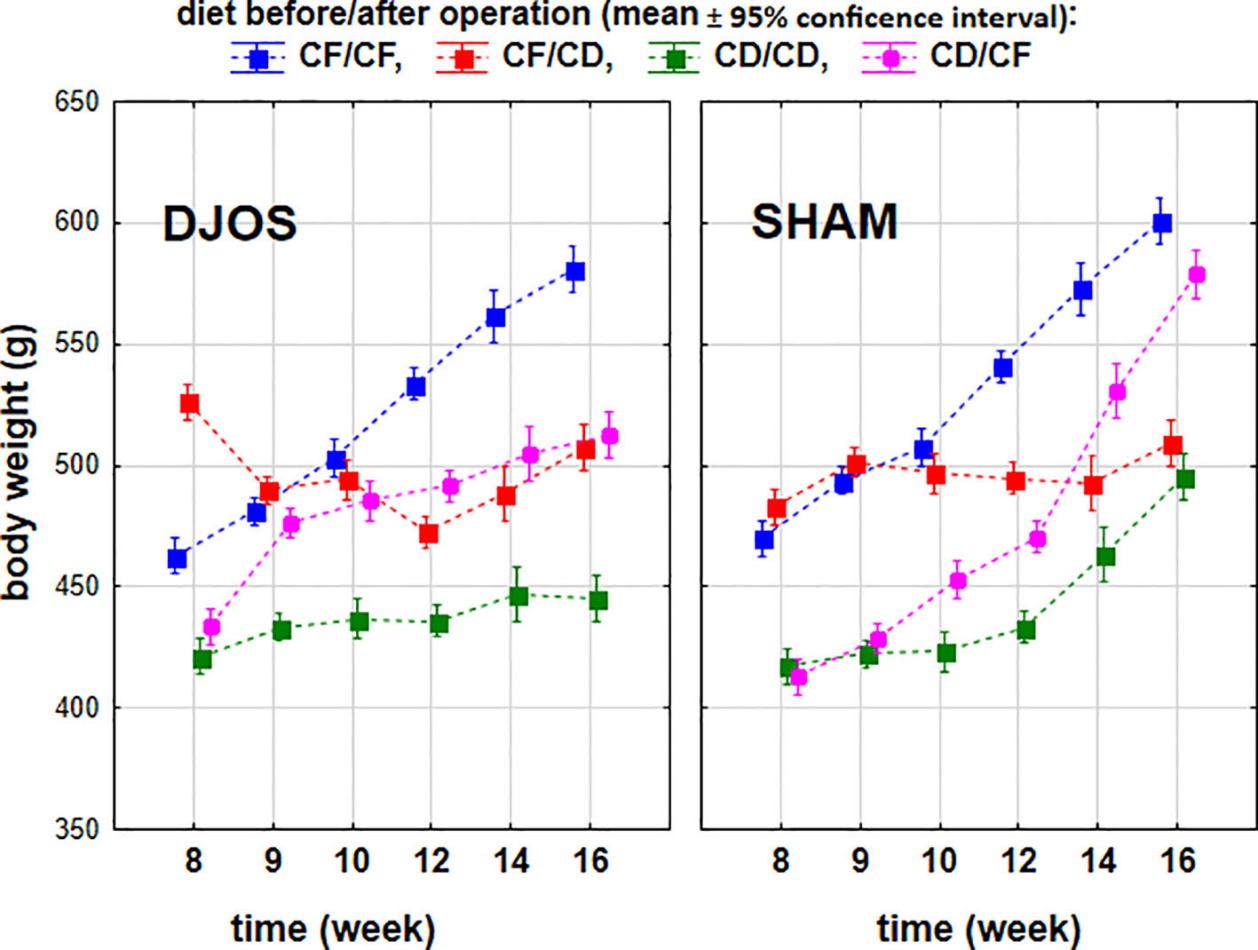
Body weight (g) of Sprague-Dawley rats (n = 56) subjected to duodenojejunal omega switch (DJOS, n = 28) surgery or control (SHAM, n = 28) surgery and different dietary protocols (CD/CD, CD/CF, CF/CD, CF/CF, n = 7 for each) during the post-surgery phase of the experiment. Legend: CD, control diet; CF, high-fat/high-sugar diet. To compare body weights within individual treatments, a two-factor ANOVA was used, accounting for the type of diet used for repeated measurements with multiple comparison analysis.

In week 16 of the study (post-surgery week 9), the rats from the CF/CF group subjected to SHAM surgery exhibited significantly higher body weights than rats subjected to DJOS surgery and fed according to the CF/CF (p < 0.01) or the CD/CD dietary protocol (p < 0.001) (**Figure 8**).

In the group of rats whose diet was changed to CD the post-operative phase of the experiment, we observed no statistically significant differences between the rats subjected to different surgical procedures (p = 0.803). In contrast, changing the diet from CD to CF resulted in higher body weights in rats after SHAM surgery than in those subjected to DJOS surgery (p < 0.001) (**Figure 8**).

The most favorable outcomes (lowest body weights) were observed in rats maintained on the CD/CD diet and subjected to DJOS surgery, whereas the least favorable outcomes were recorded in the CF/CF diet combined with SHAM surgery (**Figure 8**).

## 4 Discussion

Fatty acids serve as the fundamental components of more complex lipids, and their composition across lipid species is frequently used for comparative analysis within a lipid class when investigating diseases and physiological disruptions in lipid metabolism (LIPID MAPS®).

The liver, being the primary organ for fatty acid metabolism, is additionally significantly affected by obesity. Obesity is frequently associated with a disruption in hepatic fatty acid metabolism, in which the liver’s ability to process fatty acids and other metabolic energy sources is compromised (Friedman et al., 2018).

In our previous studies (Stygar et al., 2019b; Poloczek et al., 2021; Kazura et al., 2023), using this experimental model, DJOS surgery improved the serum lipid profile (Kazura et al., 2023) in a manner that paralleled the enhancement of glucose tolerance (Poloczek et al., 2021) and was only partly explained by modest reductions in body mass (Stygar et al., 2019b), with the dietary regimen (CD vs. HFS, control diet vs. high-fat, high-sugar diet) remaining a major determinant of the outcome. Specifically, serum total cholesterol and triglyceride concentrations increased progressively from CD/CD to HFS/HFS in both DJOS- and SHAM-operated rats, with the lowest values in CD/CD and the highest in HFS/HFS. Within each dietary scheme, however, cholesterol and triglycerides were consistently lower in DJOS than in SHAM animals (for example, total cholesterol approximately 35–55 mg/dL vs. 41–77 mg/dL, and triglycerides 49–59 mg/dL vs. 61–149 mg/dL, respectively). In addition, HDL levels after DJOS increased from CD/CD to HFS/CD (approximately 16 to 23 mg/dL), whereas SHAM animals showed a less favorable HDL pattern, with very low values in CD/CD and HFS/HFS (Kazura et al., 2023).

These lipid findings are concordant with our previously reported OGTT (oral glucose tolerance test) data (Poloczek et al., 2021), in which DJOS consistently reduced glycemia and AUC_OGGT_ compared with SHAM, particularly in HFS-based groups (CD/HFS, HFS/CD, HFS/HFS), while the lowest AUC (area under curve) values were always observed in CD/CD irrespective of the surgical procedure. The type of diet exerted a strong, operation-independent effect on AUC, with HFS/HFS yielding the highest and CD/CD the lowest values (Poloczek et al., 2021). Under the same dietary allocation, DJOS produced only moderate body-weight reductions compared with SHAM (e.g., CD/CD ~361 vs. 382 g; HFS/HFS ~435 vs. 473 g), and all HFS-exposed groups remained clearly obese; nevertheless, in these heaviest groups DJOS still improved both lipid profile (lower cholesterol/TG) and OGTT relative to SHAM (Stygar et al., 2019b).

Taken together, in HFS/HFS and HFS/CD groups DJOS markedly lowered cholesterol (Kazura et al., 2023) and triglycerides and improved glucose tolerance (Poloczek et al., 2021) despite relatively small differences in body weight (Stygar et al., 2019b), indicating a substantial, relatively weight-independent metabolic action, likely involving altered nutrient flow, gut–liver signaling and reduced oxidative stress. In contrast, in CD/CD groups, where body mass was similar between DJOS and SHAM, lipid profile and OGTT were also most similar, suggesting that when the diet is favorable the incremental effect of DJOS is limited. The overall pattern is consistent: the more adverse the diet (HFS), the more strongly DJOS attenuates both dyslipidemia and glucose intolerance compared with SHAM, although complete normalization is not achieved, underscoring the dominant impact of HFS and the importance of dietary control after surgery. Thus, DJOS in this model concurrently improves lipid profile (lower total cholesterol and triglycerides, partial HDL improvement) and glucose tolerance, with only a moderate effect on body mass, particularly under HFS conditions, supporting the concept that the beneficial changes in glycemia and lipidemia are largely driven by modulation of the gut–liver axis and oxidative status rather than by weight loss alone.

The relationship between dietary fat intake and metabolic disorders is complex and context-dependent, with studies reporting positive, negative, or neutral effects for specific fatty acids, even within the same class (SFAs, MUFAs, PUFAs, and trans fats) (Sivri and Akdevelioğlu, 2025). Medium-chain fatty acids (MCFAs), particularly capric acid (C10:0), have been associated with improved lipid and glucose metabolism and reduced risk of type 2 diabetes. Capric acid enhances glucose tolerance via GPR84-mediated GLP-1 secretion, as demonstrated in both STC-1 cell models and mice, and confers resistance to high-fat diet–induced obesity (Nonaka et al., 2022). Diets enriched in MCFAs, compared with lard, improve metabolic parameters and increase plasma GLP-1 levels, with capric acid showing the most pronounced effects.

Capric acid, abundant in tropical oils such as coconut oil (Shrestha et al., 2015), also exhibits antioxidant and anti-inflammatory properties. In mice, MCFA-rich diets reduce lipid peroxidation (TBARS), preserve paraoxonase 1 activity, and attenuate IL-6 and IL-10 production by splenocytes, mitigating oxidative stress– and inflammation-related obesity complications (Martínez-Galán et al., 2021). Furthermore, MCFAs influence gut microbiota composition, suppressing pathogenic species (e.g., *Vibrio cholerae*, *Salmonella typhi*, enterotoxigenic *Escherichia coli*) while promoting beneficial commensals, including *Lactobacillus* spp. of the Enterococcaceae family (Zentek et al., 2012).

Animal studies indicate that a diet rich in coconut oil or medium-chain fatty acids preserves insulin and glucose tolerance compared to diets rich in saturated or unsaturated long-chain lipids (Wein et al., 2009). Replacing roughly 30 g (5% of calories) of long-chain saturated fatty acids with medium-chain fatty acids in the diet has been demonstrated to avert insulin resistance (Lundsgaard et al., 2021). Feeding rodents diets with 42–60% fat from coconut oil or MCFAs oil for 4–12 weeks preserved insulin and glucose tolerance comparable to that of a control diet (Buettner et al., 2006; Wein et al., 2009).

High-saturated-fat diets promote glucose intolerance and increase susceptibility to diabetes through multiple interrelated mechanisms. A central role is played by palmitic acid, which induces oxidative stress and inflammation, disrupts insulin signaling via DAG- and ceramide-mediated lipotoxicity, and exacerbates insulin resistance through PKC activation and IL-1β release. In addition, palmitic acid impairs mitochondrial and endoplasmic reticulum function, promotes pancreatic β-cell apoptosis, enhances reactive oxygen species production, and activates toll-like receptor 4 signaling, thereby further amplifying inflammatory and metabolic dysfunction (Palomer et al., 2018). Significantly excessive intake of SFAs, including palmitic acid, promotes the accumulation of hepatic and visceral fat and enhanced gluconeogenesis in the cells, compared with PUFAs (Rosqvist et al., 2014). Overall, even-chain SFAs intake is associated with the risk of type 2 diabetes (Forouhi et al., 2014), but the magnitude of this risk depends on the specific sources of saturated fat. Saturated fat in butter and cheese is associated with an increased risk of diabetes, whereas consumption of full-fat yoghurt is associated with a lower risk (Guasch-Ferré et al., 2017). In diet-induced obese rats, myristic (C14:0) and lauric (C12:0) acids were strongly associated with markers of insulin resistance and metabolic syndrome, including body weight gain, glucose levels, and HOMA-IR (Cao et al., 2008). Notably, although palmitoleic acid (C16:1) is generally considered a lipokine linked to improved insulin sensitivity, its serum levels were increased under high-calorie feeding and positively correlated with adverse metabolic outcomes in this model, indicating a context-dependent role in metabolic regulation (Cao et al., 2008; Sampey et al., 2012).

Monounsaturated fatty acids, which are abundant in foods such as nuts, avocado, and olive oil and constitute a major component of the Mediterranean diet, are associated with anti-inflammatory, cardiovascular, and metabolic benefits, including improved lipid profiles and reduced cancer risk (Bos et al., 2010). Experimental studies in mice further demonstrate that long-term consumption of a MUFA-rich diet promotes an anti-inflammatory profile, characterized by increased IL-4 and IL-10 levels and reduced pro-inflammatory cytokines compared with an SFA-rich diet (Tamer et al., 2020).

Oleic acid (C18:1) plays a protective role against insulin resistance and type 2 diabetes by reducing glucolipotoxicity and oxidative stress, improving β-cell function and survival, and enhancing insulin sensitivity through adiponectin upregulation (Palomer et al., 2018). As a direct PPARα agonist, oleate suppresses NF-κB signaling and hepatic inflammation, contributing to the anti-inflammatory effects of monounsaturated fatty acids (Patil et al., 2019). Long-term observational data further indicate that regular olive oil consumption lowers the risk of type 2 diabetes (Guasch-Ferré et al., 2015), while mechanistic studies suggest that oleic acid preserves AMPK activity, similarly to metformin, and that an elevated palmitic-to-oleic acid ratio may increase diabetes risk, particularly in women (Palomer et al., 2018). Since oleic acid is one of the most abundant monounsaturated fatty acids (MUFAs) and is widely present in animal fats and vegetable oils characteristic of the cafeteria (high-fat/high-sugar) diet (de la Garza et al., 2023), its elevated dietary intake under CF conditions may have contributed to increased hepatic accumulation. Therefore, the reduction in hepatic oleic acid concentration observed in SHAM-operated rats following post-surgical dietary improvement (CF/CF vs. CF/CD) can be interpreted as a consequence of decreased exogenous supply resulting from the transition to the control diet.

Humans are unable to synthesize linoleic acid (LA; omega-6; C18:2 n-6) and α-linolenic acid (ALA; C18:3 n-3), which must be obtained from the diet. ALA serves as a precursor to EPA (C20:5 n-3), DPA (C22:5 n-3), and DHA (C22:6 n-3), making dietary intake critical for their physiological effects (Ratnayake and Galli, 2009). Maintaining an n-6:n-3 ratio of 1:1–2:1 supports homeostasis, whereas Western diets with ratios of 10:1–20:1 may promote obesity and related metabolic disorders through excessive arachidonic acid–mediated proinflammatory signaling (Simopoulos, 2016). Moderate LA intake (~5–7% of total energy) is associated with a 9–17% reduction in type 2 diabetes risk (Zong et al., 2019), though specific n-6 fatty acids such as gamma-linolenic acid (GLA) may increase risk via gut microbiota perturbations (Miao et al., 2020).

n-3 fatty acids enhance insulin sensitivity by promoting β-oxidation, modulating Akt and AMPK signaling, and directly affecting β-cell function via PPARγ, GPR40, and GPR120. In adipose tissue, GPR120 activation increases GLUT4 translocation and glucose uptake, while n-3 fatty acids inhibit lipogenesis, modulate gut microbiota, and reduce proinflammatory cytokines. Nonetheless, epidemiological evidence is inconsistent: high total n-3 intake has been associated with increased type 2 diabetes risk in some cohorts (Dow et al., 2016), whereas lower intake of PUFAs, including ALA, may be protective (Neuenschwander et al., 2020). Systematic reviews suggest that overall, n-3, n-6, and total PUFAs exert minimal effects on diabetes prevention or management (Brown et al., 2019).

Currently, the number of direct research studies investigating the effects of bariatric procedures, such as Roux-en-Y gastric bypass (RYGB), sleeve gastrectomy (SG), or one-anastomosis gastric bypass (OAGB), on plasma FAs is scarce. However, different bariatric procedures yield distinct hepatic FA profiles, as they are based on distinct mechanisms.

Bariatric surgery exerts rapid improvements in insulin resistance, independent of weight loss, and significantly modulates lipid metabolism and circulating fatty acid composition, potentially influencing medium-chain fatty acids such as capric acid (C10:0). In comparative studies, intermittent fasting with caloric restriction (IF-CR) altered metabolite profiles more markedly than sleeve gastrectomy (SG), with downregulation of ketone bodies (acetoacetate, β-hydroxybutyrate) and certain saturated and monounsaturated long-chain fatty acids, and upregulation of gluconeogenic precursors (alanine, glycerate), proline, branched-chain amino acids (valine, leucine, isoleucine), and microbiota-derived metabolites, including short- and medium-chain fatty acids (butyric, caproic, caprylic acids) and indole-3-acetate (IAA), a key mediator of gut microbiota–host interactions (Haran et al., 2023). Consistently, our study demonstrated elevated hepatic capric acid concentrations in DJOS-operated rats fed a high-fat/high-sugar diet, highlighting the interplay between surgical intervention, diet, and MCFA metabolism.

Bariatric surgery has been shown to alter hepatic long-chain fatty acid (LCFA) profiles. Following Roux-en-Y gastric bypass (RYGB), myristic acid (C14:0) levels in the liver were significantly reduced, while pentadecanoic acid (C15:0), an odd-chain saturated fatty acid, also exhibited post-surgical changes, reflecting shifts in hepatic fatty acid composition (Zizmare et al., 2022). Moreover, Roux-en-Y gastric bypass (RYGB) surgery is also associated with long-term alterations in lipid profiles. At nine months post-surgery, concentrations of seven non-esterified fatty acids (NEFAs)—myristate (C14:0), palmitate (C16:0), palmitoleate (C16:1), stearate (C18:0), oleate (C18:1c9), linoleate (C18:2), and arachidonate (C20:4)—were significantly reduced. Overall levels of total NEFAs, as well as total saturated (SFA) and unsaturated fatty acids (UFA), decreased, while the n-3:n-6 ratio remained unchanged (Hierons et al., 2022).

Twelve months after laparoscopic sleeve gastrectomy (LSG), patients exhibited significantly lower serum levels of several saturated fatty acids (SFAs), including caprylic (C8:0), capric (C10:0), lauric (C12:0), myristic (C14:0), palmitic (C16:0), and tricosanoic (C23:0) acids, as well as reduced monounsaturated fatty acids (MUFAs) such as myristoleic (C14:1), palmitoleic (C16:1), and elaidic (C18:1 trans n-9). Overall, total SFA and MUFA percentages decreased, while the relative proportion of polyunsaturated fatty acids (PUFAs) increased, particularly linoleic acid, arachidonic acid, and DHA (docosahexaenoic acid) (Wrzosek et al., 2022).

The impact of bariatric surgery on polyunsaturated fatty acids (PUFAs) remains inconsistent across studies. Some reports indicate reductions in DHA and arachidonic acid (AA) post-surgery, while linoleic acid (LA), α-linolenic acid (ALA), and the bioactive n-3 PUFA EPA may transiently decrease for up to six months before gradually returning to preoperative levels by approximately 12 months. Malabsorptive procedures, such as Roux-en-Y gastric bypass (RYGB) and biliopancreatic diversion with duodenal switch (BPD/DS), appear to exert a more pronounced effect on circulating PUFA concentrations compared with restrictive interventions, including laparoscopic adjustable gastric banding (LAGB) and laparoscopic sleeve gastrectomy (LSG) (Middleton et al., 2022).

Our DJOS model (preserving pylorus, excluding foregut) uniquely elevates MCFA (C10:0) and selected LCFA/VLCFA under the CF diet, unlike RYGB, decreasing SFA/NEFA (Hierons et al., 2022) or LSG increasing PUFA% (Wrzosek et al., 2022). These procedure-specific changes in β-oxidation, hindgut signaling, and malabsorption justify focused investigation of DJOS mechanisms.

The present findings are part of a long-term study investigating the effects of diet and bariatric procedures, including duodenojejunal omega switch (DJOS) and ileal transposition (IT), on metabolic and biochemical outcomes in animal models. To date, evidence indicates that diet composition is a primary driver of metabolic dysfunction. In Sprague–Dawley rats, high-fat (HF) and high-fat/high-sugar (HFS) diets consistently induce obesity, glucose intolerance, insulin resistance, systemic inflammation, and oxidative stress (Skrzep-Poloczek et al., 2018; Stygar et al., 2018c; Stygar et al., 2018a; Stygar et al., 2018b; Stygar et al., 2019a; Stygar et al., 2019b; Poloczek et al., 2021). These metabolic disturbances are associated with dysregulation of incretin hormones and insulin secretion (Stygar et al., 2018a; Stygar et al., 2019a), elevated circulating adipokines and hepatokines (FABP4, leptin, chemerin, fetuin-A, and fetuin-B), increased inflammatory markers including CRP and pentraxin 3 (PTX3), and activation of cellular stress responses, as evidenced by upregulation of HSP70 and HSP90 (Stygar et al., 2018c; Stygar et al., 2018a; Stygar et al., 2018b; Stygar et al., 2019b).

Duodeno-jejunal omega switch surgery exerts broad metabolic effects that extend beyond body weight reduction. It improves glucose tolerance and modulates incretin secretion even under continued high-fat feeding, highlighting the role of gut-derived hormonal signaling (Stygar et al., 2018c). In addition, DJOS normalizes key enzymes of glucose metabolism in the liver and skeletal muscle, such as GSK-3α, PYGM, PYGL, and PFK, indicating improved insulin sensitivity (Stygar et al., 2019a), and alters hepatic expression of metabolic regulators such as RBP4 and FGF21 (Stygar et al., 2018b). These changes are accompanied by reduced levels of adipokines, hepatokines, and inflammatory markers, including leptin, FABP4, chemerin, fetuins, GDF-15, and PTX3 (Stygar et al., 2018a; Stygar et al., 2018b; Poloczek et al., 2021), as well as marked attenuation of oxidative stress and cellular stress responses in metabolically active tissues (Skrzep-Poloczek et al., 2018; Stygar et al., 2019b). Across studies, the most favorable metabolic profile is observed when DJOS is combined with a post-operative control diet (Stygar et al., 2018c; Poloczek et al., 2021).

In contrast, IT primarily affects metabolic regulation through endocrine mechanisms. In rats with diet-induced obesity and glucose intolerance, IT enhances incretin and insulin secretion and improves glucose homeostasis despite ongoing high-fat feeding (Sawczyn et al., 2019). Ileal transposition also reduces circulating ANGPTL8 and PTX3 levels, suggesting modulation of pathways linking glucose metabolism with inflammation (Sawczyn et al., 2021).

In summary, high-fat dietary patterns are the primary drivers of metabolic deterioration, whereas bariatric procedures partially reverse these changes through complementary mechanisms. DJOS induces multi-level metabolic reprogramming involving hormonal, enzymatic, inflammatory, oxidative, and gene-regulatory pathways, strongly modulated by diet composition. By contrast, IT predominantly improves glucose metabolism through incretin-driven mechanisms and selective anti-inflammatory effects.

Taken together, these findings indicate that while dietary intervention remains essential for optimizing hepatic lipid metabolism, DJOS induces surgery-specific metabolic adaptations that may complement and augment the beneficial effects of dietary modification.

From a translational perspective, these findings suggest that the bariatric procedures may influence fatty acid metabolism beyond weight loss alone. These effects highlight the importance of integrating surgical strategy with postoperative nutritional management to maximize metabolic outcomes and may support personalized dietary recommendations following bariatric procedures.

We acknowledge that our study has certain limitations. One of the limitations is the relatively small sample size, although the distribution of groups is well-balanced. Since our study has a cross-sectional design, it has inherent limitations related to the nature of the study, where causality cannot be elucidated. Nevertheless, our careful design and the use of gas chromatography-mass spectrometry for FA determination are important strengths in this study.

## 5 Conclusions

This study demonstrates that dietary regimen is the primary determinant of hepatic fatty acid (FA) composition, while bariatric surgery contributes to more specific, procedure-related metabolic changes. The diet’s quality substantially influenced liver lipid profiles, as postoperative dietary improvement in SHAM-operated rats reduced hepatic concentrations of several saturated and unsaturated fatty acids.

In parallel, duodenojejunal omega switch (DJOS) surgery was associated with distinct alterations in hepatic FA composition. DJOS led to higher hepatic levels of selected medium-chain, long-chain, and very long-chain fatty acids. Notably, the increased hepatic concentration of caproic acid following DJOS suggests modified handling of medium-chain fatty acids, which may contribute to favorable metabolic and anti-inflammatory effects.

## Data Availability

The raw data supporting the conclusions of this article will be made available by the authors, without undue reservation.
